# To What Extent Is Hb_A1c_ Associated with Glycemic Variability in Patients with Type 1 Diabetes? A Retrospective, Noninterventional Study

**DOI:** 10.3390/jcm13020450

**Published:** 2024-01-13

**Authors:** Sandra Lazar, Ioana Ionita, Delia Reurean-Pintilei, Romulus Timar, Silvia Ana Luca, Bogdan Timar

**Affiliations:** 1 First Department of Internal Medicine, “Victor Babes” University of Medicine and Pharmacy, 300041 Timisoara, Romania; sandra.lazar@umft.ro; 2 Department of Hematology, Emergency Municipal Hospital, 300254 Timisoara, Romania; 3 Centre for Molecular Research in Nephrology and Vascular Disease, “Victor Babes” University of Medicine and Pharmacy, 300041 Timisoara, Romania; drdeliapintilei@gmail.com (D.R.-P.); timar.romulus@umft.ro (R.T.); silvia.luca0@yahoo.com (S.A.L.); bogdan.timar@umft.ro (B.T.); 4 Multidisciplinary Research Center for Malignant Hematological Diseases (CCMHM), “Victor Babes” University of Medicine and Pharmacy, 300041 Timisoara, Romania; 5 Department of Diabetes, Nutrition and Metabolic Diseases, Consultmed Medical Centre, 700544 Iasi, Romania; 6 Second Department of Internal Medicine, “Victor Babes” University of Medicine and Pharmacy, 300041 Timisoara, Romania; 7 Department of Diabetes, Nutrition and Metabolic Diseases, “Pius Brinzeu” Emergency Hospital, 300723 Timisoara, Romania; 8 Department of Cardiology, “Victor Babes” University of Medicine and Pharmacy, 300041 Timisoara, Romania

**Keywords:** glycemic variability, time in range, coefficient of variation, standard deviation, type 1 diabetes, diabetes management

## Abstract

Background: Glycemic variability (GV) is a novel parameter used in evaluating the quality of diabetes management. Current guidelines recommend the use of GV indexes alongside the traditional parameter to evaluate glycemic control: hemoglobin A1c (HbA1c). This study aims to evaluate the extent to which HbA1c explains the GV phenomena in patients with Type 1 diabetes (T1DM). Methods: In 147 patients with T1DM, associations between HbA1c and several GV indexes were analyzed. Results: Patients with an HbA1c < 7% had a lower median standard deviation of glycemia (60 vs. 48; *p* < 0.001), a lower coefficient of variation (34.1 vs. 38.0; *p* < 0.001), and a significantly increased median time in range (78 vs. 58; *p* < 0.001). HbA1c was positively correlated with the coefficient of variation (r = 0.349; *p* < 0.001) and the standard deviation (r = 0.656; *p* < 0.001) but reversely correlated with a lower time in range (r = −0.637; *p* < 0.001). Conclusions: HbA1c only partially explains the GV phenomena in patients with T1DM. The HbA1c value is associated more strongly with the time in range and standard deviation than with the coefficient of variation.

## 1. Introduction

Hemoglobin A1c (HbA1c) is a widely used parameter for evaluating glycemic control and considered the primary tool for assessing diabetes management [1,2]. Thus, until recently, the clinical standard practice for assessing therapeutic success among patients with Diabetes Mellitus (DM) was expressed as an HbA1c value below 7% [3]. 

However, HbA1c represents only a weighted average of the glycemic values from the past 3 months maximum, an average that can be obtained either from distant or close values. Furthermore, the measurement of HbA1c does not provide any information regarding the range or amplitude of the glycemic value oscillations, which can mask the true glycemic status and thus may cause a failure to identify increased risks of acute and chronic DM complications [4,5,6]. Therefore, a patient can have multiple hypoglycemic or hyperglycemic episodes but with a good average of glycemic values using a target level of HbA1c [7].

These oscillations in glycemic values are more important in Type 1 Diabetes Mellitus (T1DM), where the balance of glycemic values is much frailer due to the absolute absence of endogenous insulin. T1DM is characterized by the lack of insulin production. Therefore, glycemic control relies on exogenous insulin administration. Since there is large individual variability in insulin requirements, which can be augmented by several factors such as meals, physical activity, stress, hormonal impairment inflammation, and infections, along with variable individual responses to insulin action, T1DM patients tend to have higher glycemic fluctuations with an increased risk of developing hypoglycemic and hyperglycemic episodes and accentuating glycemic instability, making it necessary to develop new indicators for quantifying glycemic oscillations [8,9,10].

A higher GV is associated with an increased risk of hypo- or hyperglycemia in patients with similar HbA1c levels. Several studies suggested that GV has an additional independent impact on the risk of both microvascular (such as diabetic neuropathy, diabetic retinopathy, and chronic kidney disease) and macrovascular (such as cardiovascular disease and cerebrovascular disease) complications associated with DM [11,12,13,14,15,16]. There are several explanations to support this statement. First, a higher GV leads to an increase in hypoglycemic episodes. The Diabetes Control and Complications Trial (DCCT) showed that the risk of occurrence for hypoglycemic episodes cannot be fully explained by HbA1c, suggesting that lowering HbA1c levels are associated with an exponential increase in the risk of developing hypoglycemic episodes [17,18]. Later studies demonstrated that GV has an additional independent role in evaluating hypoglycemic risk [17,18]. By increasing oxidative stress and inflammation markers, both long-term and recurrent short-term hypoglycemia contribute to the occurrence of diabetic complications such as cardiovascular disease, diabetic retinopathy, diabetic neuropathy, and cognitive impairment, along with increasing the risk of mortality [19,20,21,22,23]. Secondly, it is known that chronic hyperglycemia makes a major contribution to the pathogenesis of diabetic comorbidities, but intermittent hypoglycemia also plays a major role in the development of associated pathologies, along with altering the immune system, leading to poor outcomes after infections [24,25]. This result emphasizes the limitations of using only HbA1c since it cannot assess short-term hyperglycemic events such as postprandial hyperglycemia. Lastly, dysglycemic events are not the only factor associated with an increased risk for diabetic comorbidities. It was also found that glycemic oscillations are associated with increased mortality in DM patients [26]. Moreover, in experimental studies, fluctuations in glycemic values were found to have a negative impact on endothelial tissue, thus leading to an increase in cardiovascular risk [27].

The introduction of continuous glycemic monitoring systems (CGMS) led to the development of a series of novel parameters aimed at the evaluation of glycemic control. CGMSs can reveal both the glycemic values and the glycemic trends of the patient in real time and thus allow the patient to improve his or her glycemia and avoid hypoglycemia, as well as to store the recorded values, thereby allowing for further analyses of time-related glycemia, including analyses of the main GV indicators, coefficient of variation (CV), and standard deviation (SD) [28,29].

GV has emerged as a valuable tool in evaluating the management of diabetes. On the one hand, GV is a vector of diabetes complications. On the other hand, it is known that a high GV represents a major burden in achieving the target values of the traditional glycemic control parameter [30]. 

CV is a derivate index used to assess GV. This index can precisely evaluate the daily variation in glycemic fluctuations. CV allows one to compare and standardize the GV in DM patients with different glycemic means. Benefitting from the advantage of a simple calculation formula, a CV below 36% is considered optimal for proper diabetes management [31,32].

SD is a widely used parameter for assessing GV. This parameter measures the dispersion of glycemic values around the mean [29] and is directly correlated with GV. Therefore, a higher SD is associated with higher GV and poorer glycemic control [33]. Moreover, SD can help identify a glycemic pattern, along with evaluating the patient’s treatment interventions, which can lead to establishing an optimal glycemic range goal. Limitations in SD correspond to the fact that glycemic values do not have a parametric distribution and cannot, therefore, accurately distinguish between minor and major glycemic oscillation [34]. However, SD remains an extensively used indicator and is the simplest tool for GV evaluation [35]. In addition, it was demonstrated that GV expressed as SD is an important predictor for macrovascular complication in DM patients with acute coronary syndrome [36].

Another valuable parameter for glycemic metrics that is more frequently used in clinical practice is time in range (TIR), which represents the percentage of time in which the glycemia value remains within a predefined range that can be reached in a safe scenario, generally between 70 and 180 mg/dL [37,38]. Optimal glycemic control is achieved when the TIR is above 70%, along with less than 4% of glycemic values below the target range, thereby minimizing the time that blood glucose values are above the target range [3,39]. Despite not being an intrinsic GV indicator, TIR offers an intuitive and reliable overview of the factors that cause disturbances in glycemic balance (such as various physical activities or types of food). 

Moreover, in current clinical guidelines, the American Diabetes Association (ADA) mentions that to achieve an optimal evaluation of glycemic control, it is necessary to analyze both the HbA1c level and the CGMS parameters. The guidelines also offer standardized recommendations regarding the values of these parameters for T1DM and for Type 2 Diabetes Mellitus patients (T2DM) [2]. 

Recent studies revealed that an increased GV was associated with an increased incidence of hyperglycemic episodes, leading to an increase in HbA1c levels [40,41]. Moreover, a stronger correlation between a lower TIR and increased HbA1c levels was hypothesized since TIR does not consider only glycemic fluctuations but also the range of the glycemic values [40,41]. Since each aforementioned parameter has its own limitations and evaluates glycemic control in a different manner, incorporating GV measurements alongside HbA1c levels in clinical practice can provide valuable insights for diabetes management. Analyzing both the weighted means of glycemic values (through HbA1c levels) and intra-day glycemic fluctuations could have a major therapeutic impact by reducing the risk of DM-associated comorbidities and increasing patient quality of life. Therefore, the main aim of this study was to determine the presence of an association between HbA1c, GV, and TIR in T1DM patients and to measure the extent of that association. 

## 2. Materials and Methods

### 2.1. Study Design and Patients

In total, 147 patients previously diagnosed with Type 1 Diabetes Mellitus (T1DM) were enrolled in this retrospective, non-interventional, multicentric, population-based, consecutive-case enrollment study. All patients attended scheduled outpatient visits either at the Diabetes Outpatient Centre of “Pius Brinzeu” Emergency Hospital, Timisoara, Romania (105 patients) or at Consultmed Clinic, Iasi, Romania (42 patients). All patients were treated with insulin for T1DM: 84 (57.1%) using basal bolus insulin therapy, combining a basal insulin analogue (Tresiba, Lantus or Toujeo) plus prandial insulin (rapid or ultrarapid analogues—Novorapid, Humalog, Fiasp or Lyumjev, respectively), and 63 (42.9%) with rapid insulin analogues (Novorapid, Humalog, Fiasp, or Lyumjev) administered using continuous insulin infusion systems (insulin pumps). In the studied cohort, 60 patients (40.8%) were men and 87 (59.2%) were women. The median age of the patients was 32 years (interquartile distance 15 years), and the median diabetes duration was 7 years (interquartile distance 10 years). Patients had a median body mass index of 22.6 kg/m^2^ (interquartile distance 6.2 kg/m^2^) and a median HbA1c of 7.0% (interquartile distance 0.9%). 

The study design and protocol were approved by the independent Ethics Committee of the “Victor Babes” University of Medicine and Pharmacy, Timisoara, Romania (No. 50 from 19 October 2021). 

### 2.2. Glycemic Variability Assessment

GV assessments were performed using continuous glucose monitoring systems among all patients and were evaluated over a period of 90 days before the study visit. In all patients, measurements were performed using Medtronic Guardian 3 or Medtronic Guardian Link systems with the same type of sensor and provider for glucose monitoring sensors. The CGM system was calibrated according to the manufacturer’s guidelines, and participants were trained in its use to minimize errors and ensure data accuracy. The GV indexes evaluated were the standard deviation (SD) of the glycemic values measured in the last 90 days and the corresponding coefficient of variation (CV) provided by the device’s standardized reports. At the same time, the Time in Range (TIR) parameter was evaluated in all patients and also provided by the device reports. Mean glucose (MG) provided the average glucose concentration over the monitoring period, while SD reflected the fluctuations around the mean value. CV, expressed as a percentage, was calculated as the SD divided by the MG and provided a normalized measure of variability. As recommended by the current management guidelines, a CV < 36% was considered a therapeutic target for the GV, considering that this value describes stable glycemic values. For TIR, the standard 70 mg/dL–180 mg/dL interval was considered the desired range of glycemia in all patients. 

### 2.3. Clinical, Biological, and Laboratory Assessments

For each participant, age, diabetes duration, type of insulin regimen, and gender were collected from the patient’s medical records. HbA1c was measured on the day of the visit using the DCCT-standardized chemiluminescence method, which is presented as percentage points. HbA1c levels were measured to provide a longer-term perspective on glycemic control. Blood samples were collected from participants at the beginning and end of the monitoring period. The HbA1c measurement was performed using the high-performance liquid chromatography (HPLC) method, which is considered the gold standard due to its high specificity and accuracy. This method quantifies the percentage of hemoglobin that is glycated, providing an average blood glucose level over the past two to three months.

The procedure involved drawing venous blood samples into EDTA-containing tubes, which were then centrifuged. Next, the plasma was separated. HbA1c levels were determined using an HPLC analyzer, which was calibrated according to the manufacturer’s instructions and operated by trained laboratory personnel. The results were reported as a percentage of total hemoglobin, and the intra-assay and inter-assay coefficients of variation were maintained below 2%, ensuring highly reliable measurements. In all patients, a HbA1c value lower than 7% was considered the desired treatment target. During the visit, height and body weight were measured, and the body mass index (BMI) was calculated using the following formula: BMI = body weight (kg)/height (m)^2^. Obesity was defined as a BMI value higher than 30 kg/m^2^.

### 2.4. Statistical Analysis

Data were collected and analyzed using the Statistical Package for Social Sciences (SPSS) version 29 (IBM Corporation, Armonk, NY, USA). The results are presented as the median values and interquartile distance (continuous variables with non-parametric distribution); mean and standard deviation (continuous variables with Gaussian distribution); or absolute frequency and percentage from the subgroup total (nominal variables). The shape of the variable distribution was evaluated using the Shapiro–Wilk method, and the homogeneity of variance was evaluated using Levene’s test. 

To evaluate the statistical significance of the differences between groups, Mann–Whitney U (comparison of medians) or chi-squared (comparison of proportions) tests were used. To evaluate the strength and direction of correlations between continuous variables, Spearman’s rho correlation coefficient was used. To evaluate the goodness of fit for logistic regression models, the Nagelkerke’s R2 coefficient was used, while the statistical significance of the model was evaluated using the method proposed by Hosmer and Lemeshow. The most discriminant point in the receiver operating characteristic analysis was determined to be Youden’s index (the value in which the sum of sensitivity and specificity of the diagnosis test is maximized). The sample size was calculated a priori to achieve the study’s main objective, i.e., a statistical power of 0.80 in parallel with a confidence level of 95%. 

In this study, a *p*-value lower than 0.05 was considered the threshold of statistical significance. 

## 3. Results

In the studied group of patients, 79 (53.7%) had an HbA1c lower than 7%, 74 (50.3%) achieved a coefficient of variability lower than 36%, and 67 (45.6%) achieved an overall TIR higher than 70% during the 90 days of evaluation. 

No significant differences regarding the use of insulin pumps vs. basal bolus insulin regimens were observed between men and women (50.0% vs. 37.9%; *p* = 0.146; chi-squared test). A higher proportion of patients using insulin pump therapy vs. basal bolus insulin therapy achieved both an overall TIR higher than 70% (54.0% vs. 39.3%; *p* = 0.077; chi-squared test) and a coefficient of variation lower than 36% (58.7% vs. 44.0%; *p* = 0.078; chi-squared test); these differences were marginally statistically significant. No significant differences in achieving the <7% HbA1c target were observed between the treatment regimens (55.6% for insulin pump users vs. 52.4% in patients treated using basal bolus insulin regimens; *p* = 0.146, chi-squared test). 

### HbA1c and Glycemic Variability

**Figure 1:**
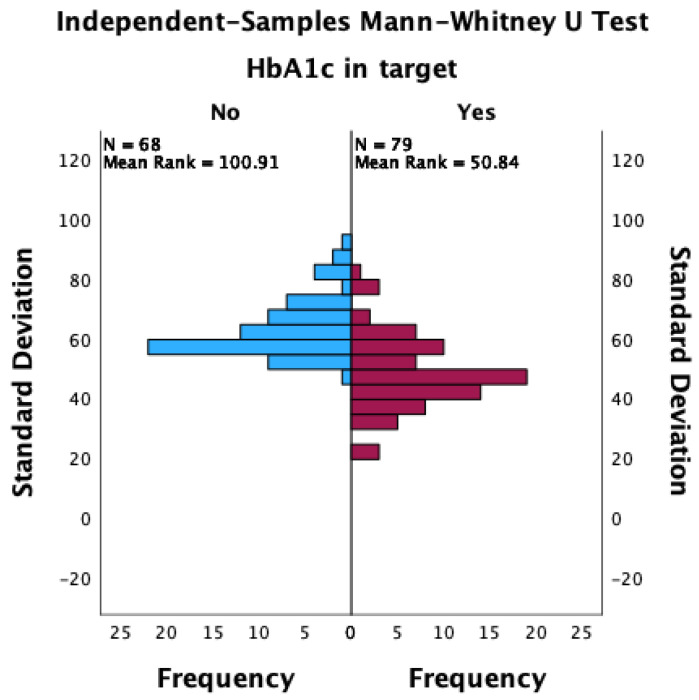
The distribution of standard deviations between patients with HbA1c values that achieved the target and those that did not achieve the target.

**Figure 2:**
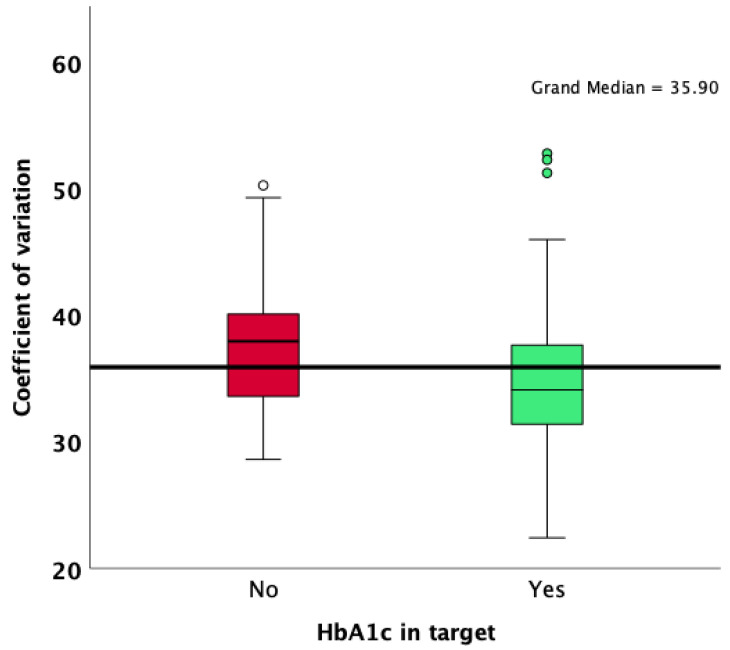
Comparison of CV results according to HbA1c target achievement.

**Figure 3:**
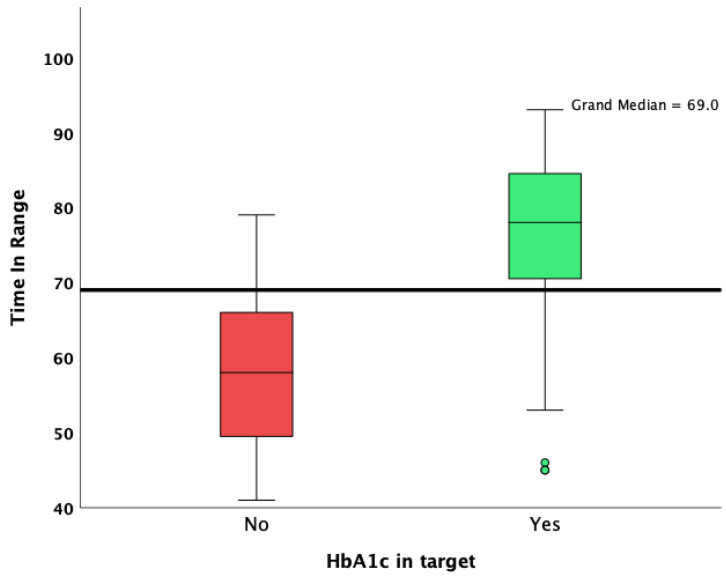
Overall TIR in patients who achieved and did not achieve the HbA1c target.

Patients with an HbA1c value lower than 7% were more likely to achieve an overall TIR higher than 70%, as well as a CV lower than 36%. Patients who achieved an HbA1c target of less than 7% had a lower median standard deviation of glycemia measured during the 90 days of observation (48 vs. 60; *p* < 0.001; Mann–Whitney U test; Figure 1), a lower CV (34.1 vs. 38.0; *p* < 0.001; Mann–Whitney U test; Figure 2), and a significantly increased median TIR (78 vs. 58; *p* < 0.001; Mann–Whitney U test; Figure 3). 

Differences in glycemic variability indexes between patients who obtained and did not obtain the HbA1c < 7% target are presented in Table 1. 

**Figure 4:**
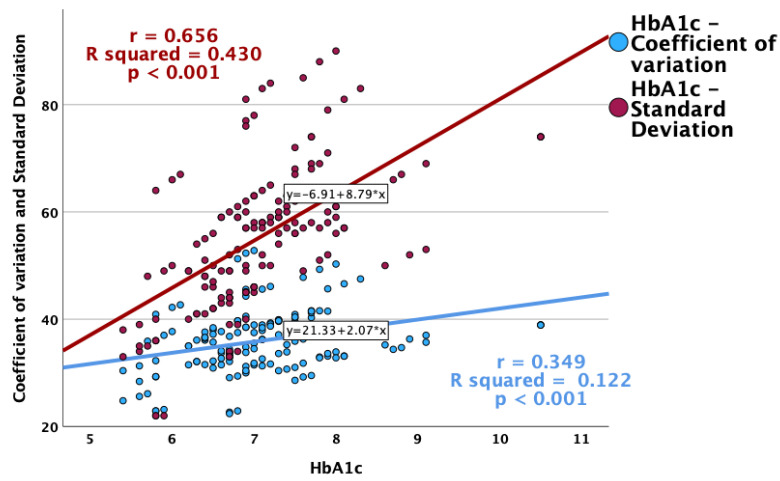
Correlations between HbA1c and glycemic variability indexes (CV and standard deviation).

**Figure 5:**
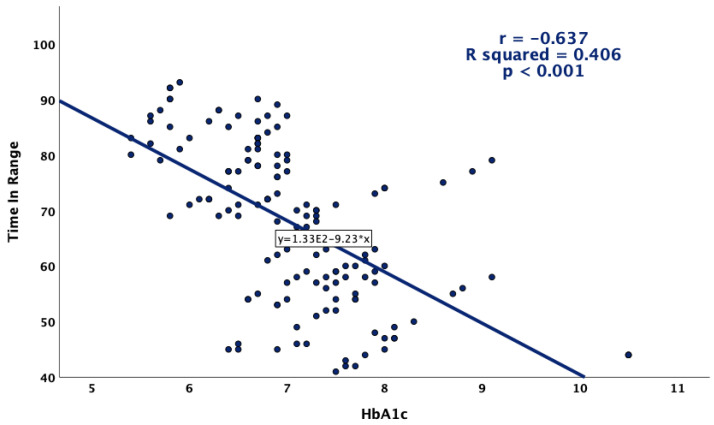
Correlation between HbA1c and TIR.

Glycemic variability was more pronounced in patients with higher HbA1c values (Figure 4). Positive and statistically significant correlations were observed between HbA1c and CV, as well as the standard deviation, while a higher HbA1c was associated with a lower overall TIR (Figure 5). 

**Figure 6:**
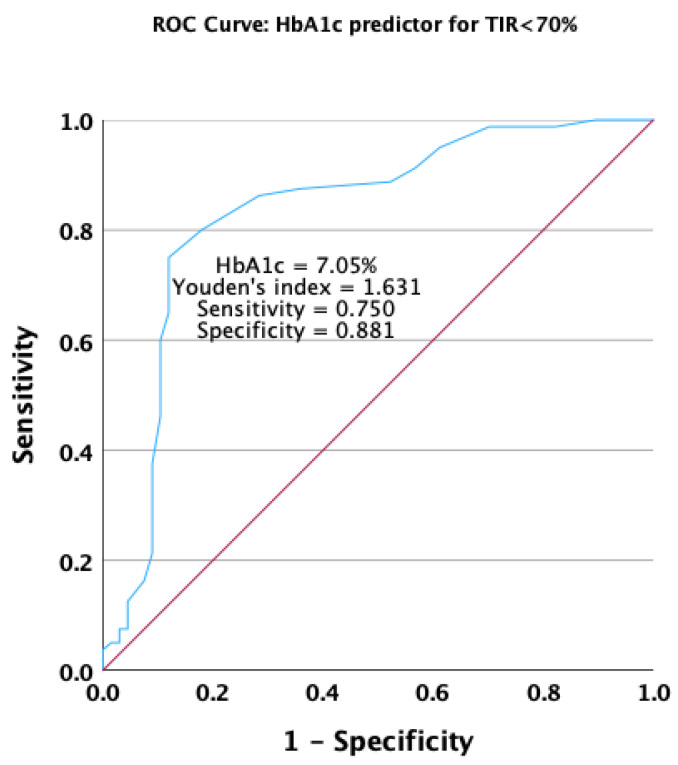
Receiver operating characteristic curve for HbA1c as a predictor for not achieving the target overall TIR.

A decrease in the HbA1c value was found to be a good predictor for achieving a TIR < 70% in the receiver operating characteristic analysis. The HbA1c value had an area under the ROC curve of 0.833 (*p* < 0.001), with a 95% confidence interval ranging from 0.762 to 0.905 (Figure 6). 

To evaluate the impact of the HbA1c value on the likelihood of achieving an overall TIR higher than 70%, a univariate logistic regression model was built with TIR target achievement as the dependent variable. This model was found to offer a good fit, with variation in HbA1c explaining 35.1% of the variation in the likelihood of achieving the TIR target (Nagelkerke R^2^ = 0.351; *p* < 0.001, Hosmer and Lemeshow test). This model demonstrated that for each 1% increase in the value of HbA1c, the likelihood of achieving an overall TIR > 70% decreased by 83.4%, leading to OR = 0.166, with a 95% confidence interval for an Exp(β) between 0.084 and 0.326. 

**Figure 7:**
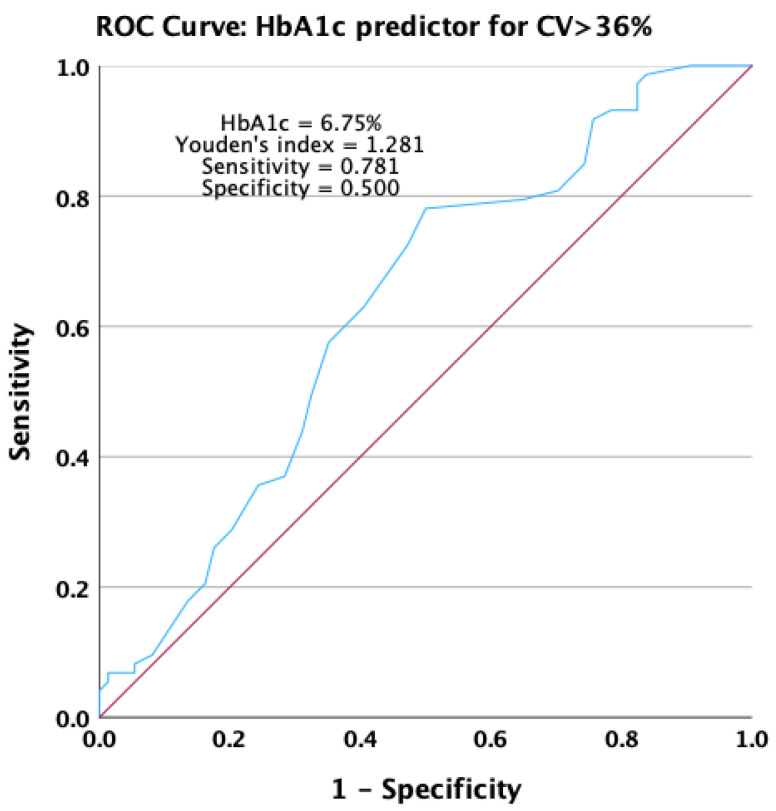
Receiver operating characteristic curve for HbA1c as a predictor for not achieving stable glycemic values.

An increase in the HbA1c value significantly predicted the occurrence of unstable glycemic values in the receiving operating characteristic analysis. HbA1c presented an area under the ROC curve of 0.631 (*p* = 0.006), with a 95% confidence interval ranging from 0.540 to 0.721 (Figure 7). 

To evaluate the impact of the HbA1c value on the likelihood of obtaining stable glycemic values, defined as a CV lower than 36%, a univariate logistic regression model was built with the CV target as the dependent variable. This model demonstrated a significant fit, with variation in HbA1c explaining 7.0% of variation in the likelihood of achieving stable glycemic values (Nagelkerke R^2^ = 0.070; *p* = 0.020, Hosmer and Lemeshow test). This model demonstrated that for each 1% increase in the value of HbA1c, the likelihood of achieving a CV < 0.36 decreased by 42.7%, leading to OR = 0.573, with a 95% confidence interval for an Exp(β) between 0.379 and 0.868. 

## 4. Discussion

The main finding of this study is that when examining the association between HbA1c and GV expressed as SD, CV, and TIR, a complex interrelationship emerges. The results suggest a strong association between the HbA1c value and the various measures of GV. Specifically, patients who obtained the target HbA1c level were associated with a lower SD value along with an optimal CV, leading to an increase in the median TIR. In contrast, patients with an HbA1c level above 7% had a more pronounced GV, with a higher SD and CV and a decrease in TIR, revealing interdependence between HbA1c and GV. 

The therapeutic responses of patients following insulin pump therapy compared with patients following basal bolus therapy with multiple insulin injections were also investigated. However, no clinical differences were observed.

The strengths of this retrospective, non-interventional study include an investigation of real-life CGMS data in a thoroughly individualized cohort of patients with T1DM over a 90-day period using the same type of CGMS, without any particular restrictions. CGMS data collection was carried out remotely through an online platform, thus limiting the time spent by the patient at the hospital. No adverse effects regarding CGMS were observed. Using a robust statistical methodology, including a comprehensive approach, a direct analysis of the researched parameters was performed. To date, this is the only study from Eastern Europe that analyzes the relationship between HbA1c and glycemic variability expressed as SD and CV.

Certain limitations of this study should be acknowledged. The study design was cross-sectional, which may have limited the ability to establish causality. Longitudinal studies are needed to further explore the relation between HbA1c and GV over time. On the other hand, patients included in the study were generally young, with a mean age of 32 and a recent onset of DM. Future studies are needed to expand this research to an older cohort of patients.

The SD of blood glucose represents one of the main GV parameters. The results of this study reveal a positive correlation between HbA1c levels and SD, suggesting that higher HbA1c values lead to an increase in glycemic fluctuations. This observation emphasizes that the measurement of DM management, with respect to the glycemic control, should not be focused only on the mean of the glycemic values but also on the dispersion of those values around the average. This association is supported by several articles [42,43,44]. A recent study that included over 600 young patients with T1DM showed a positive correlation between SD and HbA1c, indicating that a higher HbA1c leads to poorer glycemic control [42]. In addition, an analysis that included over 400 patients with T1DM and Type 2 Diabetes Mellitus (T2DM), stratified in two groups, showed a significant interaction between SD and HbA1c, an effect that was more noticeable with higher HbA1c values [43]. 

In the present study, more than half of the patients had an optimal HbA1c level. In this group, most patients achieved a TIR > 70%, showing a significant association between HbA1c and TIR. For all study group patients, the TIR values were set to between 70 and 180 mg/dL in the same manner, according to the guidelines. This finding suggests that individuals with the target value of HbA1c generally spent more time within the target glucose range. This result is consistent with previous literature data, highlighting the linear relationship between HbA1c and TIR. A literature review that analyzed 18 clinical articles revealed a strong correlation between TIR and HbA1c, indicating that for every 10% change in the TIR value, a 0.8% change in HbA1c could be predicted [45]. 

Indeed, it was hypothesized that a lower TIR could be a predictive factor for developing DM complications. A study using the Diabetes Control and Complications Trial (DCCT) dataset developed in 2019 reported that each 10% drop in TIR increases the risk of diabetic retinopathy progression by 64% and the development of microalbuminuria by 40% [46]. 

On the other hand, this study identified patients who, despite having an HbA1c above 7%, reached an optimal level of TIR. This result emphasizes the limitations of both TIR and HbA1c as independent markers for glycemic control, demonstrating that a comprehensive assessment that incorporates real-time measurements is needed.

Lastly, the results of this study revealed a negative correlation between CV and HbA1c. CV can be used to determine the day-to-day fluctuations in glycemic values and is the main parameter for assessing GV [31,47]. Moreover, these results suggest that for each 1% increase in the HbA1c value, the probability of achieving an optimal CV decreases by 42.7%. This result indicates that individuals with a lower HbA1c tend to have a lower GV, underscoring the importance of measuring glycemic fluctuations to assess glycemic control. Moreover, it is presumed that patients with a higher CV tend to have more hypoglycemic episodes than patients with an ideal CV. A study that included 76 patients demonstrated that patients with a CV above 36% tend to have more hypoglycemic episodes, even though they had higher HbA1c levels compared to the levels among patients with a CV lower than 36% [32].

## 5. Conclusions

The HbA1c value was associated with all three analyzed GV indexes, TIR, CV, and SD. The relationship between HbA1 and TIR or SD was stronger compared to the association between HbA1c and CV. 

The HbA1 value only partially explained the GV phenomena in patients with T1DM. Thus, for a comprehensive evaluation of diabetes management, GV indexes should always be used in parallel with traditional risk biomarkers such as HbA1 and fasting or post-prandial glycemia. 

Further research is needed in patients with T1DM to evaluate factors associated with increased GV. 

HbA1c remains a viable parameter for assessing glycemic control. However, considering its limitations in describing GV, a comprehensive real-time analysis of glycemic fluctuations is needed in all T1DM patients to achieve adequate diabetes management. 

## Figures and Tables

**Table 1:** Comparison of glycemic variability parameters between patients who achieved and did not achieve the HbA1c < 7% target.

	HbA1c < 7% (*n* = 79)	HbA1c ≥ 7% (*n* = 68)	*p*-Value
**TIR ≥ 70% target achievement ^a^**	59 (74.7%)	8 (11.8%)	<0.001 *
**CV ≤ 36% target achievement ^a^**	48 (60.8%)	26 (38.2%)	0.005 *
**TIR (percentage points) ^b^**	78 [70 to 85]	58 [50 to 66]	<0.001 *
**CV (percentage points) ^b^**	34.1 [31.3 to 37.7]	38.0 [33.6 to 40.1]	<0.001 *
**Standard Deviation of Glycemia ^b^**	48 [41 to 57]	60 [57 to 69]	<0.001 *

^a^ Nominal variables. The results are presented as absolute frequencies and the percentage of the subgroup total. The p-value was calculated using a chi-squared test. ^b^ Continuous variables with non-parametric distribution. Results are presented as the median values and interquartile range. The p-value was calculated using a Mann–Whitney U test. * Differences between groups are statistically significant at α < 0.05 threshold.

## Data Availability

The data presented in this study are available on request from the corresponding author. The data are not publicly available due to local privacy and data protection regulations.
